# Pertussis-Associated Pneumonia in Infants and Children From Low- and Middle-Income Countries Participating in the PERCH Study

**DOI:** 10.1093/cid/ciw546

**Published:** 2016-11-02

**Authors:** Breanna Barger-Kamate, Maria Deloria Knoll, E. Wangeci Kagucia, Christine Prosperi, Henry C. Baggett, W. Abdullah Brooks, Daniel R. Feikin, Laura L. Hammitt, Stephen R. C. Howie, Orin S. Levine, Shabir A. Madhi, J. Anthony G. Scott, Donald M. Thea, Tussanee Amornintapichet, Trevor P. Anderson, Juliet O. Awori, Vicky L. Baillie, James Chipeta, Andrea N. DeLuca, Amanda J. Driscoll, Doli Goswami, Melissa M. Higdon, Lokman Hossain, Ruth A. Karron, Susan Maloney, David P. Moore, Susan C. Morpeth, Lawrence Mwananyanda, Ogochukwu Ofordile, Emmanuel Olutunde, Daniel E. Park, Samba O. Sow, Milagritos D. Tapia, David R. Murdoch, Katherine L. O'Brien, Karen L. Kotloff

**Affiliations:** 1 Departmentof Pediatrics, Division of Emergency Medicine, Johns Hopkins School of Medicine, Baltimore, Maryland; 2 Spokane Emergency Physicians, Washington; 3 Department of International Health, International Vaccine Access Center, Johns Hopkins Bloomberg School of Public Health, Baltimore, Maryland; 4 Global Disease Detection Center, Thailand Ministry of Public Health–US Centers for Disease Control and Prevention Collaboration, Nonthaburi; 5 Division of Global Health Protection, Center for Global Health, Centers for Disease Control and Prevention, Atlanta, Georgia; 6 Department of International Health, Johns Hopkins Bloomberg School of Public Health, Baltimore, Maryland; 7 International Centre for Diarrhoeal Disease Research, Bangladesh (icddr,b), Dhaka and Matlab; 8 Division of Viral Diseases, National Center for Immunizations and Respiratory Diseases, Centers for Disease Control and Prevention, Atlanta, Georgia; 9 Kenya Medical Research Institute–Wellcome Trust Research Programme, Kilifi; 10 Medical Research Council Unit, Basse, The Gambia; 11 Department of Paediatrics, University of Auckland; 12 Centre for International Health, University of Otago, Dunedin, New Zealand; 13 Bill & Melinda Gates Foundation, Seattle, Washington; 14 Medical Research Council, Respiratory and Meningeal Pathogens Research Unit; 15 Department of Science and Technology/National Research Foundation, Vaccine Preventable Diseases Unit, University of the Witwatersrand, Johannesburg, South Africa; 16 London School of Hygiene and Tropical Medicine, United Kingdom; 17 Center for Global Health and Development, Boston University School of Public Health, Massachusetts; 18 Sa Kaeo Provincial Hospital, Thailand Ministry of Health, Sa Kaeo; 19 Microbiology Unit, Canterbury Health Laboratories, Christchurch, New Zealand; 20 Department of Paediatrics and Child Health, University of Zambia School of Medicine; 21 University Teaching Hospital, Lusaka, Zambia; 22 Department of Epidemiology; 23 Department of International Health, Center for Immunization Research, Johns Hopkins Bloomberg School of Public Health, Baltimore, Maryland; 24 Division of Global HIV and Tuberculosis, Center for Global Health, Centers for Disease Control and Prevention, Atlanta, Georgia; 25 Microbiology Laboratory, Middlemore Hospital, Counties Manukau District Health Board, Auckland, New Zealand; 26 Milken Institute School of Public Health, Department of Epidemiology and Biostatistics, George Washington University, Washington D.C.; 27 Centre pour le Développement des Vaccins, Bamako, Mali; 28 Division of Infectious Disease and Tropical Pediatrics, Department of Pediatrics, Center for Vaccine Development, Institute of Global Health, University of Maryland School of Medicine, Baltimore; 29 Department of Pathology, University of Otago, Christchurch, New Zealand

**Keywords:** pertussis, infant, pneumonia, whooping cough, vaccination

## Abstract

***Background.*** Few data exist describing pertussis epidemiology among infants and children in low- and middle-income countries to guide preventive strategies.

***Methods.*** Children 1–59 months of age hospitalized with World Health Organization–defined severe or very severe pneumonia in 7 African and Asian countries and similarly aged community controls were enrolled in the Pneumonia Etiology Research for Child Health study. They underwent a standardized clinical evaluation and provided nasopharyngeal and oropharyngeal swabs and induced sputum (cases only) for *Bordetella pertussis* polymerase chain reaction. Risk factors and pertussis-associated clinical findings were identified.

***Results.*** *Bordetella pertussis* was detected in 53 of 4200 (1.3%) cases and 11 of 5196 (0.2%) controls. In the age stratum 1–5 months, 40 (2.3% of 1721) cases were positive, all from African sites, as were 8 (0.5% of 1617) controls. Pertussis-positive African cases 1–5 months old, compared to controls, were more often human immunodeficiency virus (HIV) uninfected-exposed (adjusted odds ratio [aOR], 2.2), unvaccinated (aOR, 3.7), underweight (aOR, 6.3), and too young to be immunized (aOR, 16.1) (all *P* ≤ .05). Compared with pertussis-negative African cases in this age group, pertussis-positive cases were younger, more likely to vomit (aOR, 2.6), to cough ≥14 days (aOR, 6.3), to have leukocyte counts >20 000 cells/µL (aOR, 4.6), and to have lymphocyte counts >10 000 cells/µL (aOR, 7.2) (all *P* ≤ .05). The case fatality ratio of pertussis-infected pneumonia cases 1–5 months of age was 12.5% (95% confidence interval, 4.2%–26.8%; 5/40); pertussis was identified in 3.7% of 137 in-hospital deaths among African cases in this age group.

***Conclusions.*** In the postneonatal period, pertussis causes a small fraction of hospitalized pneumonia cases and deaths; however, case fatality is substantial. The propensity to infect unvaccinated infants and those at risk for insufficient immunity (too young to be vaccinated, premature, HIV-infected/exposed) suggests that the role for maternal vaccination should be considered along with efforts to reduce exposure to risk factors and to optimize childhood pertussis vaccination coverage.

Most deaths from pertussis occur in developing countries and among children during the first weeks or months of life [1]. The World Health Organization (WHO) estimated that in 2013, *Bordetella pertussis* caused approximately 60 257 deaths in children <5 years of age [2]. However, there is uncertainty regarding these estimates, largely due to the paucity of data from the areas where most deaths occur. The variable clinical presentation of pertussis, especially in young infants, and the cost and complexity of diagnostic assays compound the difficulty in ascertaining accurate estimates. Protecting young infants from pertussis is considered a priority [3]. To achieve this goal, robust data on pertussis case-fatality and risk factors are needed to guide prioritization of control strategies and public health interventions.

The Pneumonia Etiology Research for Child Health (PERCH) Study, described in detail elsewhere [4], provided an opportunity to examine the clinical and epidemiologic characteristics of pertussis among infants and young children hospitalized with WHO-defined severe and very severe pneumonia in 7 developing countries. Here we report the pertussis findings among all PERCH-enrolled children, with more detailed analyses of those most at risk for pertussis, infants <6 months of age.

## METHODS

### Participants

Between August 2011 and January 2014, each site enrolled children 1–59 months old into PERCH during 24 consecutive months. Study sites included Dhaka and Matlab, Bangladesh; Basse, The Gambia; Kilifi, Kenya; Bamako, Mali; Soweto, South Africa; Sa Kaeo and Nakhon Phanom, Thailand; and Lusaka, Zambia. Sites, selected to represent pneumonia epidemiology in low- and middle-income settings, contained a mix of urban and rural populations, high and low human immunodeficiency virus (HIV) prevalence rates, and varying infant mortality [4]. Identification and enrollment of cases hospitalized with severe and very severe pneumonia have been described [5]. Severe pneumonia was defined as presence of cough or difficulty breathing plus lower chest wall indrawing; very severe pneumonia was defined as cough or difficulty breathing, plus at least 1 danger sign (ie, central cyanosis, difficulty breastfeeding/drinking, vomiting everything, convulsions, lethargy, reduced consciousness, or head nodding) [6]. Cases were excluded if they were hospitalized within the previous 14 days, discharged as a PERCH case within the past 30 days, did not reside in the study catchment area, or recovered from lower chest wall indrawing following bronchodilator therapy for those with wheeze.

Control children were enrolled year round, randomly selected from the community serving as the catchment area for cases, and frequency-matched to cases by enrollment date and age within the following strata: 28 days to 5 months, 6–11 months, 12–23 months, and 24–59 months, as described [5]. Those found to have WHO-defined severe or very severe pneumonia were ineligible and referred for medical care; those with nonsevere respiratory illness were eligible for enrollment.

The study protocol was approved by the institutional review board or ethical review committee overseeing each site and at the Johns Hopkins Bloomberg School of Public Health. Parents or guardians of participants provided written informed consent.

### Data Collection

Cases and controls underwent a standardized clinical assessment that included review of pertinent medical history, respiratory findings, WHO danger signs, comorbidities, and possible risk factors (eg, lack of immunization and breastfeeding). Height, weight, and pulse oximetry were measured during a focused physical examination. Thirty days after enrollment, cases were reevaluated to ascertain vital status and clinical outcomes. We defined underweight as weight for age at enrollment <−2 SD of the median age- and sex-specific WHO reference; prematurity as gestational age <37 weeks by parental report; low birth weight as <2.5 kg or small size at birth by parental report; HIV-infected as either positive virological test or HIV seropositive if >12 months old. We defined HIV uninfected-exposed as an infant or child with a negative virologic test for HIV who had evidence of HIV exposure, defined as HIV seropositive (if <12 months of age), or seronegative with a maternal history of HIV infection (for all ages), with the caveat that maternal exposure must be confirmed by maternal serology for seronegative infants aged <7 months.

Pertussis vaccine administration dates were collected from vaccination cards, when available, or by maternal history when the card was unavailable. Whole-cell pertussis vaccine was used at all sites except South Africa where acellular pertussis vaccine was used. The recommended schedule was 6, 10, and 14 weeks at all sites except The Gambia (2, 3, and 4 months) and Thailand (2, 4, and 6 months), with a booster at 18 months in South Africa (Supplementary Table 1*A*). Vaccination status was determined by the number of age-appropriate vaccine doses that were received at least 2 weeks before enrollment or 2 weeks before cough onset (for children with cough); the child was deemed undervaccinated if fewer doses were received than recommended for age, and unvaccinated if no doses were received in this time frame. Children were considered too young for vaccination if they were unvaccinated and were enrolled or had onset of cough before 8 weeks (5 sites) or 10 weeks (The Gambia and Thailand) of age. The child's age used in this definition was based on the child's age 2 weeks before enrollment or 2 weeks before cough onset (for children with cough).

Chest radiographs (CXRs) were performed on cases and were read and adjudicated according to WHO-standardized interpretation procedures by trained readers who were blinded to the clinical or laboratory findings [7]. CXR positivity was defined as consolidation and/or other infiltrate.

A blood sample was collected for a complete blood count (cases only) and HIV antibody testing (cases [all sites except Bangladesh] and controls [at all sites except Thailand, Bangladesh, and The Gambia]). Using standardized methods, induced sputum (IS) was collected from cases only, and a flocked nasopharyngeal (NP) swab (flexible minitip, Copan) and a rayon oropharyngeal (OP) swab specimen were collected from each case and control; the NP and OP swabs from each child were placed into the same vial with 3 mL of universal transport media (UTM; Copan) [8, 9].

NP/OP and IS specimens were tested in-country by multiplex polymerase chain reaction (PCR) for 33 pathogens, including the *B. pertussis* insertion sequence IS*481* (FTD Resp 33, Fast-track Diagnostics, Sliema, Malta) [10]. When >1 IS specimen was available from a child, only the first was included; results were considered analyzable regardless of sputum quality indicators such as density of epithelial cells by microscopy [11]. Standard PCR curves were generated every 3 months during the testing phase to calculate pathogen load from PCR cycle threshold values. All samples positive for *B. pertussis* by multiplex PCR underwent uniplex *B. pertussis* IS*481* and *Bordetella holmesii recA* PCR assays at Canterbury Health Laboratories (Christchurch, New Zealand) [12]. Children with at least 1 sample that tested positive for *B. pertussis* IS*481* and negative for *B. holmesii recA* were regarded as having pertussis infection.

### Statistical Analysis

Data collected at each site were entered into an electronic data capture system maintained by the Data Coordinating Center (Emmes Corporation, Rockville, Maryland). To be analyzable as a case or a control in this analysis, at least 1 respiratory sample had to be tested. The Kruskal–Wallis test was used to compare quantitative PCR data between pertussis-positive cases and controls to evaluate whether higher median density was associated with clinical infection. To compare continuous and binary characteristics between pertussis-positive and -negative cases and controls, as applicable, multiple logistic regression adjusted for site, and age where appropriate, was used. Analyses involving case fatality ratios were restricted to sites with pertussis-positive cases. Two-sided *P* values <.05 were considered significant. Analyses were performed using Stata software version 12.1 and SAS version 9.4.

## RESULTS

### Overall Pertussis Positivity

A total of 4232 cases and 5325 control children were enrolled in PERCH during the 24-month study period at each site; data from 4200 cases (99.2%) and 5196 controls (97.6%) were considered analyzable for pertussis. Pertussis was detected by PCR in 53 cases (1.3%; range by site, 0%–2.5%) and 11 control children (0.2%; range by site, 0%–0.6%) (adjusted odds ratio [aOR] for cases vs controls: 5.1; 95% confidence interval [CI], 2.6–9.8; *P* < .01) (Table 1). An additional 6 cases and 5 controls had samples that were PCR positive for *B. holmesii*, and were not included in this analysis. Pertussis-positive cases were detected at all sites except Thailand; positive controls were found in Mali, South Africa, and Thailand. The preponderance of pertussis cases (52/53 [98.1%]) and controls (8/11 [72.7%]) came from Africa. Adjusting for differences in the age distribution of cases, continent (Africa vs Asia: aOR, 8.8; *P* = .03) was significantly associated with pertussis positivity. Within Africa, site was not a significant determinant of positivity. Among the 300 PERCH cases 1–59 months of age who died in-hospital and had pertussis testing at all sites combined, 8 (2.7%) tested positive for pertussis.

**Table 1.** Study Participants Enrolled and Tested for *Bordetella pertussis*, Percentage Positive by Age and Site, and Case Fatality Ratio Among Pertussis-Positive Cases

Age Group	GAM	KEN	MAL	SAF	ZAM	BAN	THA	Total
1–5 mo								
No. enrolled								
Case	259	209	307	458	327	136	38	1734
Control	199	234	247	365	310	221	91	1667
No. with analyzable specimen								
Case	256	208	307	455	321	136	38	1721
Control	189	231	247	363	275	221	91	1617
Reason excluded^a^								
Case	A:1, D:1, E:1	B:1	None	C:2, E:1	B:4, E:2	None	None	A:1, B:5, C:2, D:1, E:4
Control	D:2, E:7; F:1	E:2, F:1	None	E:2	E:35	None	None	D:2, E:46, F:2
No. *Bp*+ (%)								
Case	3 (1.2)	2 (1.0)	8 (2.6)	18 (4.0)	9 (2.8)	0	0	40 (2.3)
Control	0	0	2 (0.8)	4 (1.1)	0	0	2 (2.2)	8 (0.5)
No. *Bp*+ in NP-OP only/IS only/both								
Case^b^	0/1/2	0/1/1	2/0/6	1/4/13	4/4/1	…	…	7/10/23
No. *Bp*+ cases who died (%)^c^								
Case	0	0	1 (12.5)	3 (16.7)	1 (11.1)	…	…	5 (12.5)
6–59 mo								
No. enrolled								
Case	379	425	367	462	290	389	186	2498
Control	455	631	478	599	376	551	568	3658
No. with analyzable specimen								
Case	370	424	366	462	282	389	186	2479
Control	432	626	477	596	335	547	566	3579
Reason excluded^a^								
Case	A:1; B:4; E:4	B:1	E:1	None	B:4; E:4	None	None	A:1; B:9; E:9
Control	D:3; E:18, F:2	E:3, F:2	E:1	E:3	E:41	E:4	E:2	D:3, E:72, F:4
No. *Bp*+ (%)								
Case	1 (0.3)	2 (0.5)	2 (0.5)	5 (1.1)	2 (0.7)	1 (0.3)	0	13 (0.5)
Control	0	0	2 (0.4)	0	0	0	1 (0.2)	3 (0.1)
No. *Bp*+ : NP-OP only/IS only/both								
Case^b^	0/1/0	0/2/0	1/0/1	1/2/2	1/1/0	0/0/1	…	3/6/4
No. *Bp*+ cases who died (%)^d^								
Case	0	0	1 (50.0)	1 (20.0)	1 (50.0)	0	…	3 (23.1)
1–59 mo								
No. enrolled								
Case	638	634	674	920	617	525	224	4232
Control	654	865	725	964	686	772	659	5325
No. *Bp*+ (%)								
Case	4 (0.6)	4 (0.6)	10 (1.5)	23 (2.5)	11 (1.8)	1 (0.2)	0 (0.0)	53 (1.3)
Control	0 (0.0)	0 (0.0)	4 (0.6)	4 (0.4)	0 (0.0)	0 (0.0)	3 (0.5)	11 (0.2)
No. *Bp*+ cases who died (%)								
Case	0 (0.0)	0 (0.0)	2 (20.0)	4 (17.4)	2 (18.2)	0 (0.0)	…	8 (15.1)

Case-control comparisons of *Bp* positivity: all *P* < .05.

Abbreviations: BAN, Bangladesh; *Bp*, *Bordetella pertussis*; GAM, The Gambia; IS, induced sputum; KEN, Kenya; MAL, Mali; NP, nasopharyngeal; NP-OP, nasopharyngeal-oropharyngeal swab; PCR, polymerase chain reaction; SAF, South Africa; THA, Thailand; ZAM, Zambia.

^a^ Reasons for exclusion: A: met exclusion criteria for IS collection and NP PCR result not available; B: died before specimen collected; C: child could not produce specimen; D: other; E: unknown reason for missing IS and/or NP PCR results; F: parent/guardian refused.

^b^ Applicable to cases only as IS not collected for controls.

^c^ Of the 35 *Bp*-positive cases aged 1–5 months discharged alive, 21 (60%) had the 30-day follow-up completed and all cases were alive.

^d^ Of the 10 *Bp*-positive cases aged 6–59 months discharged alive, 9 (90%) had the 30-day follow-up completed and all cases were alive.

Cases 1–5 months of age were significantly more likely to be pertussis positive compared with cases aged 6–59 months (2.3% vs 0.5%; aOR, 4.4; 95% CI, 2.4–8.4; *P* < .01); similarly, controls aged 1–5 months were more likely to be pertussis positive than their 6- to 59-month-old counterparts (0.5% vs 0.1%; aOR, 5.9; 95% CI, 1.6–22.3; *P* = .01) (Table 1). Among the 43 pertussis-positive cases with both NP/OP and IS specimens available, 14 were positive by IS but not NP/OP (32.6%), whereas 2 were positive in the NP/OP but not IS (4.7%); 27 (62.8%) were positive by both. Similar results were seen when restricting to cases aged 1–5 months and when restricting to children with high-quality IS specimen (defined as <10 squamous epithelial cells per low-power field) [11].

**Figure 1.**
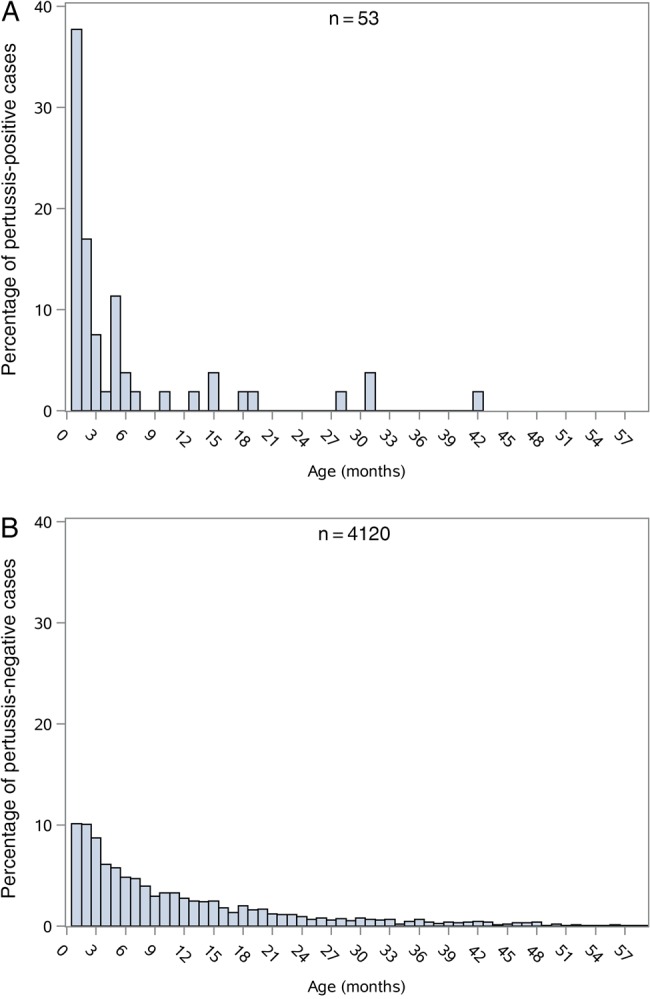
Cumulative age distribution of children with severe or very severe pneumonia by pertussis infection status as determined by polymerase chain reaction (PCR) results. *A*, Children with a positive PCR result for pertussis (n = 53). *B*, Children whose PCR results were negative (n = 4120). Pertussis-positive children were significantly younger than pertussis-negative children (median, 2 vs 7 months; *P* = .01, logistic regression adjusted for site, restricted to sites in Africa and Bangladesh where positive cases were found).

### Pertussis Among Cases and Controls 1–5 Months of Age

No pertussis-positive cases aged 1–5 months were identified at the 2 Asian sites where the proportion of controls undervaccinated for age was lower (12.5%) compared with African sites (23.5%; *P* < .0001; Table 2). Thus, to identify potential risk factors for pertussis-associated hospitalized severe and very severe pneumonia, we compared pertussis-positive cases to controls (regardless of pertussis status) only at African sites. Pertussis-positive cases were significantly younger than controls (mean, 10.3 vs 15.8 weeks; *P* < .01) and were more likely to be HIV uninfected-exposed (31.6% vs 15.2%; aOR, 2.2; 95% CI, .99–4.76; *P* = .053), underweight (35.0% vs 7.6%; aOR, 6.3; 95% CI, 3.0–13.0; *P* < .01), unvaccinated (66.7% vs 21.5%; aOR, 3.7; 95% CI, 1.5–9.2; *P* < .01), and too young to be immunized (66.7% vs 14.8%; aOR, 16.1; 95% CI, 4.1–62.6; *P* < .001) compared to controls (Table 2). Most (9/14 [64.3%]) of the underweight children were considered either premature or low birth weight; however, the association between underweight and pertussis case status remained significant after adjusting for prematurity and low birth weight. Pertussis-positive cases were more likely to have never been breastfed compared with controls (25.0% vs 9.0%; aOR, 2.5; 95% CI, 1.0–6.0; *P* = .05), but this association was no longer significant when HIV infection/exposure was included in the model (OR, 1.82; *P* = .24). Most pertussis-positive cases (26/40 [66.7%]) were unvaccinated, all because they were age-ineligible 2 weeks before cough onset (Table 2)*.* Pertussis-positive cases had a trend toward higher median density of *B. pertussis* than pertussis-positive controls (*P* = .07; Table 3; Supplementary Figure 1).

**Table 2.** Characteristics of Cases and Controls Aged 1–5 Months by Pertussis Status

Characteristic	Cases, No. (%)			Pertussis-Positive vs -Negative Cases^a^		Controls, No. (%)			Pertussis-Positive Cases vs Controls^b^	
	Pertussis Positive (n = 40)	Pertussis Negative (n = 1507)	Pertussis Negative (n = 174)	aOR^c^	*P* Value^c^	All Africa (n = 1305)	All Asia (n = 312)	Pertussis Positive (n = 8)	aOR^c^	*P* Value^c^
	Africa	Africa	Asia	Africa		Africa	Asia	Mali, South Africa, Thailand	Africa	
Age, wk, mean (SD)	10.3 (6.6)	12.9 (6.0)	14.2 (5.8)	…	.012	15.8 (5.9)	18.2 (5.0)	12.6 (7.2)	…	<.0001**
≤3 mo old	33 (82.5)	1078 (71.5)	114 (65.5)	1.78	.17	684 (52.4)	107 (34.3)*	5 (62.5)	3.87	.001**
Female	15 (37.5)	652 (43.3)	56 (32.2)	0.71	.31	657 (50.3)	154 (49.4)	4 (50.0)	0.58	.10
Never breastfed	10 (25.0)	175 (11.7)	8 (4.6)	1.84	.15	117 (9.0)	4 (1.3)*	0 (0.0)	2.47^d^	.05**
Underweight^e^	14 (35.0)	388 (25.8)	36 (20.7)	1.72	.12	98 (7.6)	22 (7.1)	2 (25.0)	6.30	<.0001**
Premature or low birth weight^f^	13 (33.3)	388 (26.0)	59 (34.1)	1.58	.19	258 (19.9)	51 (16.4)	0 (0.0)	2.02	.052
HIV-infected^g^	3 (7.5)	109 (7.2)	1 (0.6)	0.81	.74	65 (5.0)	0 (0.0)*	0 (0.0)	1.05	.93
HIV uninfected-exposed^h^	12 (31.6)	244 (18.5)	0 (0.0)	1.43	.38	166 (15.2)	0 (0.0)*	1 (12.5)	2.17	.053
Pertussis vaccination^i^										
Total										
Missing Vaccination data	1 (2.5)	43 (2.9)	3 (1.7)	0.82	.85	18 (1.4)	1 (0.3)	1 (12.5)	1.30	.80
Immunization record available	31 (79.5)	1255 (85.7)	155 (90.6)	0.35	.09	1184 (92.1)	306 (98.4)*	5 (71.4)	0.19	.0002**
Undervaccinated for age (any dose)	3 (7.7)	221 (15.1)	14 (8.2)	0.45	.18	302 (23.5)	39 (12.5)*	0 (0.0)	…	…
0 doses										
Total not vaccinated	26 (66.7)	583 (39.8)	56 (32.8)	2.82	.02	276 (21.5)	30 (9.7)	2 (28.6)	3.69	.005**
Too young	26 (66.7)	502 (34.3)	51 (29.8)	8.09	.001	190 (14.8)	25 (8.0)	2 (28.6)	16.06	<.0001**
Undervaccinated for age	0 (0.0)	81 (5.5)	5 (2.9)	…	…	86 (6.7)	5 (1.6)	0 (0.0)	…	…
1 dose										
Total 1 dose	6 (15.4)	414 (28.3)	54 (31.6)	…	…	337 (26.2)	74 (23.8)	3 (42.9)	…	…
Undervaccinated for age	1 (2.6)	65 (4.4)	8 (4.7)	…	…	89 (6.9)	9 (2.9)	0 (0.0)	…	…
2 doses										
Total 2 doses	3 (7.7)	290 (19.8)	31 (18.1)	…	…	359 (27.9)	100 (32.2)	1 (14.3)	…	…
Undervaccinated for age	2 (5.1)	75 (5.1)	1 (0.6)	…	…	127 (9.9)	25 (8.0)	0 (0.0)	…	…
3 doses										
Total 3 doses	4 (10.3)	177 (12.1)	30 (17.5)	…	…	315 (24.5)	107 (34.4)	1 (14.3)	…	…

Abbreviations: aOR, adjusted odds ratio; BAN, Bangladesh; GAM, The Gambia; HIV, human immunodeficiency virus; KEN, Kenya; MAL, Mali; OR, odds ratio; SAF, South Africa; SD, standard deviation; THA, Thailand; ZAM, Zambia.

^a^ Pertussis-positive cases compared to negative cases, restricted to Africa.

^b^ Pertussis-positive cases compared to all controls (regardless of pertussis status), restricted to Africa.

^c^ Odds ratios and *P* values from logistic regression models adjusted for site. Odds ratio for “underweight,” “premature or low birth weight,” “total not vaccinated,” and “too young” adjusted for site and age in weeks.

^d^ All 10 pertussis-positive cases who were never breastfed were from South Africa; 8 of 10 were HIV exposed. When HIV exposure is included in the model, lack of breastfeeding is no longer significantly associated with pertussis case status, compared to African controls (OR, 1.82; *P* = .24). Due to limited numbers, we cannot evaluate the association between breastfeeding and pertussis case status in the absence of HIV exposure.

^e^ Underweight defined as weight for age <−2 SD of the median age-/sex-specific World Health Organization reference. Remained independently associated with pertussis case status, compared to African controls, after adjusting for age, site, and prematurity or low birth weight (OR, 6.13; *P* < .0001).

^f^ Prematurity was defined as gestational age <37 weeks by parental report. Low birth weight was defined as <2.5 kg or small size at birth by parental report. No longer associated with pertussis case status, compared to African controls, after adjusting for age, site, and underweight (OR, 1.18; *P* = .68).

^g^ HIV-infected defined as detectable viral load or HIV seropositive if >12 months old.

^h^ HIV uninfected-exposed defined as an infant or child with a negative virologic test for HIV who had evidence of HIV exposure, defined as HIV seropositive (if <12 months of age), or seronegative with a maternal history of HIV infection (for all ages), with the caveat that maternal exposure must be confirmed by maternal serology for seronegative infants aged <7 months.

^i^ ‘Total not vaccinated’ based on number of doses received at 2 weeks prior to enrollment (for children without cough) or at 2 weeks prior to start of cough (for children with cough). ‘Too young’ defined as zero doses and age two weeks prior to enrollment or age two weeks prior to cough onset ≤8 weeks (KEN, MAL, ZAM, SAF, BAN) or ≤10 weeks (GAM, THA). ‘Under-vaccinated for age’ defined as the following: Age >8, >12 or >16 weeks with zero, one or two doses, respectively (KEN, MAL, ZAM, SAF, BAN); Age >10, >16 or >18 weeks with zero, one or two doses, respectively (GAM); Age >10, >18 or >26 weeks with zero, one or two doses, respectively (THA). Age based on age two weeks prior to enrollment or two weeks prior to cough onset.

* Significant (*P* < .05) difference comparing controls from Africa vs Asia (regardless of pertussis status) using logistics regression models or Fisher exact test; “underweight,” “total not vaccinated,” and “too young” adjusted for age in weeks.

**P ≤ .05.

**Table 3.** Association of Clinical, Laboratory, and Radiographic Findings With Pertussis Status Among Cases and Controls Aged 1–5 Months

Characteristic	Cases^a^				Controls^b^			
	Pertussis Positive (n = 40)	Pertussis Negative (n = 1507)	Pertussis Positive vs Negative		Pertussis Positive (n = 8)	Pertussis Negative (n = 693)	Pertussis Positive vs Negative	
	No. (%)	No. (%)	aOR^c^	*P* Value^c^	No. (%)	No. (%)	aOR^c^	*P* Value^c^
	Africa				Mali, South Africa, Thailand			
Runny nose, by report	12 (30.0)	552 (36.6)	0.69	.33	2 (28.6)	82 (11.9)	2.87	.28
Cough^d^	40 (100.0)	1437 (95.4)	…	.26	2 (25.0)	68 (9.8)	3.58	.14
Cough ≥ 7 d	18 (48.7)	228 (16.3)	5.0	<.0001*	0 (0.0)	10 (15.2)	…	…
Cough ≥ 14 d	10 (27.0)	77 (5.5)	6.3	<.0001*	0 (0.0)	1 (1.5)	…	…
Duration of cough, d, median (IQR)^e^	5 (3–14)	3 (2–5)	…	.004*	4 (3–5)	3 (2–5)	…	.56
Fever^d^	29 (72.5)	1168 (77.5)	1.02	.97	2 (25.0)	13 (1.9)	19.45	.004*
Vomiting^d^	14 (35.0)	266 (17.7)	2.55	.006*	1 (14.3)	1 (0.1)	151.50	.0009*
Unable to feed^d^	9 (22.5)	234 (15.5)	1.54	.27	0 (0.0)	0 (0.0)	…	…
Tachypnea^f^	28 (70.0)	1172 (78.7)	0.63	.20	4 (50.0)	108 (16.9)	…	…
Hypoxia^g^	26 (65.0)	721 (48.0)	1.49	.29	…	…	…	…
Stridor	1 (2.5)	34 (2.3)	0.95	.96	…	…	…	…
Grunting	7 (18.4)	351 (23.4)	0.62	.33	…	…	…	…
Nasal flaring	32 (80.0)	1060 (70.5)	1.38	.44	…	…	…	…
Deep breathing	2 (5.0)	194 (12.9)	0.38	.19	…	…	…	…
Audible wheeze	0 (0.0)	104 (6.9)	…	.11	…	…	…	…
CXR positive	23 (59.0)	664 (47.0)	1.50	.23	…	…	…	…
WBC, × 1000 cells/µL, median (IQR)	19.6 (14.2–34.1)	12.6 (9.1–16.7)	…	<.0001*	…	…	…	…
WBC ≥ 20 ×10^3^ cells/µL	18 (46.2)	216 (15.2)	4.6	<.0001*	…	…	…	…
Lymphocyte count ×10^3^ cells/µL, median (IQR)	10.5 (6.1–21.5)	5.9 (4.1–8.2)	…	<.0001*	…	…	…	…
Lymphocyte count ≥ 10 ×10^3^ cells/µL	20 (52.6)	179 (12.9)	7.2	<.0001*	…	…	…	…
Pertussis NP/OP PCR log copies/mL, median (IQR)^h^	7.4 (6.2–8.4)	…	…	…	5.2 (3.8–7.7)	…	…	…

“…” Represents variables that were not assessed among controls (hypoxia, stridor, grunting, nasal flaring, deep breathing, audible wheeze) or were measured among too few controls to allow meaningful comparison (WBC, lymphocytes).

Abbreviations: aOR, adjusted odds ratio; CXR, chest radiograph; IQR, interquartile range; NP/OP, nasopharyngeal/oropharyngeal; PCR, polymerase chain reaction; WBC, white blood cell.

^a^ Analysis restricted to sites with at least 1 pertussis-positive case (Kenya, The Gambia, Zambia, Mali, and South Africa).

^b^ Restricted to sites with at least 1 pertussis-positive control (Mali, South Africa, and Thailand).

^c^ Odds ratios and *P* values from logistic regression models adjusted for site. After adjusting the WBC and lymphocyte counts for age, there was a minor change in the odds ratios (WBC, 4.8; lymphocyte, 7.7), but no change in *P* value.

^d^ By history and/or physical examination. For fever, 12.5% of pertussis positive cases aged 1–5 months had documented fever; the remaining were based on reported fever.

^e^ Restricted to children with cough by history and/or physical examination. Duration in days.

^f^ Respiratory rate >60 breaths/minute if aged <2 months, >50 breaths/minute if aged 2–5 months.

^g^ A child was considered to be hypoxic if (1) a room air pulse oximetry reading indicated oxygen saturation <90% at the 2 sites at elevation (Zambia and South Africa) or <92% at all other sites, or (2) a room air oxygen saturation reading was not available and the child was on oxygen.

^h^ Median pertussis PCR log copies/mL not significantly different comparing pertussis-positive cases to pertussis-positive controls (*P* = .07, Kruskal–Wallis test).

*P ≤ .05.

We also performed case-case comparisons at the African sites to identify factors that might distinguish pertussis-positive from negative cases of severe or very severe pneumonia. Compared to cases testing negative, pertussis-positive cases were significantly younger, more likely to be unvaccinated (66.7% vs 39.8%; aOR, 2.8; *P* = .02; Figure 1; Table 2) and more likely to have findings classically associated with pertussis disease (prolonged cough longer than 7 or 14 days, no audible wheeze, vomiting, leukocytosis ≥20 000 cells/µL, and lymphocytosis ≥10 000 cells/µL; Table 3). Fifty-nine percent of pertussis-positive cases and 47.0% of pertussis-negative cases had radiographic pneumonia (*P* = .23).

A small fraction (1.1% [8/701]) of controls aged 1–5 months in Mali, South Africa, and Thailand tested positive for pertussis; none were detected at the other sites. Compared to pertussis-negative controls at those sites in this age strata, pertussis-positive controls were significantly more likely to have fever, vomiting, or tachypnea (all *P* < .05; Table 3; Supplementary Table 2), conceivably manifestations of clinically mild pertussis.

### Pertussis Mortality in Cases 1–5 Months of Age

The in-hospital case fatality ratio of pertussis-positive cases aged 1–5 months (12.5%; 95% CI, 4.2%–26.8% [5/40]) was not significantly different than that of pertussis-negative cases (8.8%; 95% CI, 7.4%–10.3% [132/1507]; *P* = .43). Deaths of pertussis-positive cases occurred in Zambia, Mali, and South Africa (Table 1). Among cases discharged alive, 30-day vital status was available for 60.0% (21/35) of pertussis-positive (with no additional deaths documented) compared with 84.6% of 1375 pertussis-negative cases. A sensitivity analysis that takes into consideration cases not tested for *B. pertussis* and the possible outcomes of cases who did not complete a 30-day follow-up visit is included in the Supplementary Appendix.

The 5 pertussis-positive deaths accounted for 3.7% of the 137 in-hospital PERCH deaths at the 5 African sites among infants 1–5 months of age who were tested for pertussis. All 5 fatal cases had an infiltrate on CXR, elevated white blood cell (WBC) count (mean, 50.2 × 10^3^ cells/µL; range, 14.2–70.0 × 10^3^ cells/µL), including 4 cases with WBC > 40 × 10^3^ cells/µL; most were very young (mean age, 13.0 weeks; range, 7–25 weeks), and 1 was HIV positive. All had multiple potential etiologic pathogens detected on both NP/OP and IS, as did most infants without pertussis infection (Supplementary Table 3). Only 1 of the pertussis-positive infants who died had received 3 doses of pertussis vaccine at least 2 weeks prior to cough onset. In contrast to the other infants who died, this infant did not manifest a leukemoid reaction (WBC count was 14.2 × 10^3^ cells/µL in the single sample tested); this infant presented with cough for 15 days, and was HIV exposed and severely underweight.

### Pertussis Among Cases and Controls 6–59 Months of Age

Among PERCH cases (n = 2477) and controls (n = 3564) 6–59 months of age with specimens tested for pertussis, 13 cases (0.5%) and 3 controls (0.1%) were positive. Compared with pertussis-negative cases, pertussis-positive cases were more likely to report cough of ≥7 days, inability to feed, leukocyte count ≥20 000 cells/µL, and lymphocyte count ≥10 000 cells/µL (all *P* < .05) (Supplementary Table 4). Three of the 13 pertussis-positive cases 6–59 months died (23.1%); 1 child was fully vaccinated, the second was HIV-positive and had received only a single pertussis vaccination, and the third child was unimmunized (clinical findings are shown in Supplementary Table 3). These 3 cases accounted for 1.9% of the 158 in-hospital deaths at the African sites in this age group.

## DISCUSSION

The PERCH study identified *B. pertussis* as a potential etiologic agent in a small fraction of severe or very severe pneumonia cases among hospitalized children 1–59 months of age in the participating low- and middle-income African and Asian countries. Pertussis was found in just 2.3% of pneumonia cases <6 months of age at all sites combined, and in 3.7% of the in-hospital deaths in this age group. Nonetheless, pertussis-infected cases suffered a considerable risk of death (12.5% among infants <6 months of age). In comparison, pertussis was identified in 0.5% of cases 6–59 months of age, 23.1% of whom died. Whereas limited access to interventions needed to manage severe respiratory infections undoubtedly contributes to mortality from severe pertussis in low resource settings, [13, 14] case-fatality rates as high as 9% have been reported for young infants with severe pertussis even in facilities with advanced critical care; these deaths are often attributed to pulmonary hypertension, necrotizing bronchopneumonia, cardiac failure, shock, and multisystem organ dysfunction [15–17].

We identified 4 risk factors for pertussis infection in PERCH cases compared to controls. The strongest factor was lack of vaccination, which increased the odds of pertussis 3.7-fold, and was unanimously attributed to the child being too young to receive vaccine. Other significant factors were young age, underweight and perinatal HIV exposure. These risk factors for severe pertussis likely reflect the commonality that young infants with insufficient immunity to pertussis are at risk for severe disease as long as pertussis is circulating in the community [18]. Accordingly, low birth weight due to prematurity, with its associated reduced transfer of specific maternal antibody, likely characterizes many of the young infants considered to be underweight in our study. Similarly, HIV uninfected-exposed newborns have been shown to acquire significantly lower levels of pertussis maternal antibody compared with unexposed infants [19], and in South Africa have a higher likelihood of pertussis-associated pneumonia requiring hospitalization [18].

We observed disparities in pertussis prevalence at the African compared with the Asian sites. This may be attributed, at least in part, to the higher rate of HIV exposure, undernutrition, low birth weight, and prematurity among African infants, all risk factors for pertussis [13]. South Africa, the site with the highest percentage of pertussis-positive cases, had the lowest (73%) coverage with 3 pertussis vaccine doses in the community as estimated from controls aged 9–18 months. Moreover, South Africa was the only site using acellular pertussis vaccine, which has lower estimates of both short-term and long-term efficacy compared with whole cell vaccine [21, 22]. Vaccine coverage was highest (>95%) in the 2 Asian sites that had no pertussis-positive cases among infants <6 months of age, which may have reduced transmission via herd immunity (Supplementary Table 1*B*).

Our study provides insights into pertussis diagnostics. First, there was strong agreement between NP/OP and IS results, although pertussis was detected more frequently from IS than from NP/OP specimens as seen elsewhere [18, 23]; IS increased detection above NP/OP alone by 48% among children with both specimens collected. If the added contribution of IS is mainly because it provides another respiratory sample, we would expect the proportion of NP-negative/IS-positive and NP-positive/IS-negative cases to be about the same, which was not the case. Second, the density of pertussis in the NP/OP, as measured in copies per milliliter, was higher in the pertussis-positive cases than the pertussis-positive controls, suggesting that greater density may be associated with disease manifestations. There have been concerns about the specificity of PCR for *B. pertussis* [24], especially the ability to also detect *B. holmesii*, a bacterium with uncertain clinical significance that has been associated with pertussis-like respiratory infections in humans [25]. Consequently, we excluded all IS*481* PCR-positive samples that also tested positive with a *B. holmesii*–specific PCR assay. In contrast to other studies that found few, if any, positive for *B. holmesii* [18, 26], we found that 17% of samples (11/66) that were positive for pertussis among the cases and controls in the multiplex PCR assay were excluded on this basis.

The highly sensitive nature of multiplex PCR leads to identification of multiple potential pathogens of uncertain significance in many children. Etiologic attribution, based on pathogens detected among both the cases and controls is the subject of a separate PERCH report. Therefore, correlation of PCR with clinical findings is of great interest in this descriptive analysis. Notably, several clinical findings observed among our pertussis cases are atypical, such as the high prevalence of fever among infants 1–5 months old, which may reflect coinfecting pathogens and/or the reliance on maternal report (only 17% of infants actually had medically documented fevers). In addition, the duration of cough was brief (<7 days) in approximately 25% of cases. Some case definitions of pertussis for infants 0–3 months old disregard cough duration; however, cough must be accompanied by classic posttussive events such as apnea, vomiting, cyanosis, seizures, or deep inspiratory whoop, features that add specificity to the diagnosis [27]. A limitation of our study is that the quality of the cough was not interrogated, and we did not inquire of parents whether vomiting was posttussive or unrelated to coughing. It is therefore reassuring that among the cases aged 1–5 months, those who were *B. pertussis* positive were significantly more likely than uninfected cases to have features highly characteristic of pertussis, such as elevated total WBC count with lymphocytosis, a cough lasting ≥7 or ≥14 days, and vomiting [16, 28–30]. Moreover, all had a clinical diagnosis of pneumonia, a diagnostic criterion for pertussis [27].

We intentionally did not estimate the vaccine effectiveness of infant pertussis vaccination. Case-control studies of vaccine effectiveness must strive to collect immunization data from all cases and controls in the same manner and aim for the greatest degree of documentation and completeness. As estimating vaccine effectiveness was not the objective of PERCH, the vaccination records were not collected in this manner. Furthermore, as controls were usually enrolled at home, where records were more often available, compared with cases, who were enrolled at hospitalization, there is a difference in the source and possibly validity of the vaccination history of cases and controls (Supplementary Table 1*A* and 1*B*).

The contribution of pertussis to pneumonia mortality in the PERCH study (2.7%), when applied to global estimates of the contribution of pneumonia to all-cause mortality in 2013 (15% in those 0–59 months of age [2]), leads to an estimate of the global pertussis contribution (0.4%) that can be compared with the current estimate of the pertussis contribution to overall childhood mortality (ie, 1% of all-cause deaths in children aged 0–59 months, inferred from an estimate of 60 000 pertussis deaths), which is based on a natural history model [2]. There are several reasons why the burden implied by PERCH might be inaccurate. The PERCH study assessed hospitalized children only and did not enroll infants in the first month of life, an age group contributing a sizeable number of the deaths among children <5 years of age, and one that is at risk for pertussis [31]. If the fraction of deaths in those 2 groups attributable to pertussis is substantially higher or lower than the observed 2.7% of deaths in PERCH, the estimate of the pertussis contribution to global deaths will increase or decrease. Furthermore, the respiratory syndrome meeting the PERCH case definition is not the only clinical presentation of pertussis; it can also present with encephalitis, cerebral hemorrhage, pulmonary hypertension [32], or apnea [33], and many cases manifest persistent cough that is paroxysmal, with or without an inspiratory whoop and posttussive apnea and/or vomiting. Finally, because the incidence of pertussis varies over time (tending to produce peaks every 2–5 years) [34], and geography [35], the study duration of 24 months at each site did not likely capture the full spectrum of disease burden for that particular site, but collectively may be largely representative as PERCH study data collection took place over a 30-month period. Therefore, the burden detected in PERCH, although likely capturing most of the serious pertussis burden, is not exhaustive [13, 36, 37].

In conclusion, the PERCH study demonstrated that pertussis causes a small fraction of severe or very severe hospitalized pneumonia and associated deaths during the postneonatal period. Nonetheless, in the African sites, approximately 1 in 10 of pertussis-infected hospitalized cases died. The risk factors we have identified point the way toward interventions that could impact this disease burden. Strategies to reduce pertussis circulation through complete infant and childhood vaccination should be emphasized, as achieving reductions in the other risk factors (eg, preterm and underweight births, undernutrition, and maternal HIV infection) is a long-term development challenge. The occurrence of pertussis in young, unvaccinated infants and in those at risk for diminished levels of maternal antibody suggests that maternal vaccination could also be a targeted strategy to further reduce disease burden. Recent observations that early second-trimester maternal acellular pertussis immunization significantly increased neonatal antibodies compared with third-trimester vaccination offer a potential strategy to improve immunity in premature infants [38]. The PERCH pertussis data contribute to the more comprehensive understanding of pertussis infection in low-resource settings and advance the prioritization of prevention strategies.

## Supplementary Data

Supplementary materials are available at http://cid.oxfordjournals.org. Consisting of data provided by the author to benefit the reader, the posted materials are not copyedited and are the sole responsibility of the author, so questions or comments should be addressed to the author.

Supplementary Data

## References

[1] Crowcroft NS, Stein C, Duclos P, Birmingham M How best to estimate the global burden of pertussis? Lancet Infect Dis 2003; 3:413–8. 12837346 10.1016/s1473-3099(03)00669-8

[2] Liu L, Oza S, Hogan D et al Global, regional, and national causes of child mortality in 2000–13, with projections to inform post-2015 priorities: an updated systematic analysis. Lancet 2015; 385:430–40. 25280870 10.1016/S0140-6736(14)61698-6

[3] Forsyth K, Plotkin S, Tan T, Wirsing von König CH Strategies to decrease pertussis transmission to infants. Pediatrics 2015; 135:e1475–82. 25963002 10.1542/peds.2014-3925

[4] Levine OS, O'Brien KL, Deloria-Knoll M et al The Pneumonia Etiology Research for Child Health Project: a 21st century childhood pneumonia etiology study. Clin Infect Dis 2012; 54:S93–101. 22403238 10.1093/cid/cir1052 PMC3297546

[5] Deloria-Knoll M, Feikin DR, Scott JAG et al Identification and selection of cases and controls in the Pneumonia Etiology Research for Child Health project. Clin Infect Dis 2012; 54:S117–23. 22403225 10.1093/cid/cir1066 PMC3297551

[6] Scott JAG, Wonodi C, Moïsi JC et al The definition of pneumonia, the assessment of severity, and clinical standardization in the Pneumonia Etiology Research for Child Health study. Clin Infect Dis 2012; 54(suppl 2):S109–16. 22403224 10.1093/cid/cir1065 PMC3297550

[7] Cherian T, Mulholland EK, Carlin JB et al Standardized interpretation of paediatric chest radiographs for the diagnosis of pneumonia in epidemiological studies. Bull World Health Organ 2005; 83:353–9. 15976876 PMC2626240

[8] Hammitt LL, Murdoch DR, Scott JAG et al Specimen collection for the diagnosis of pediatric pneumonia. Clin Infect Dis 2012; 54(suppl 2):S132–9. 22403227 10.1093/cid/cir1068 PMC3693496

[9] Grant LR, Hammitt LL, Murdoch DR, O'Brien KL, Scott JA Procedures for collection of induced sputum specimens from children. Clin Infect Dis 2012; 54:S140–5. 22403228 10.1093/cid/cir1069 PMC3297553

[10] Murdoch DR, O'Brien KL, Driscoll AJ, Karron RA, Bhat N Laboratory methods for determining pneumonia etiology in children. Clin Infect Dis 2012; 54(suppl 2):S146–52. 22403229 10.1093/cid/cir1073

[11] Murdoch D, Morpeth S, Hammitt L Microscopic analysis and quality assessment of induced sputum from children with pneumonia in the PERCH Study. Clin Infect Dis 2017 In press. 10.1093/cid/cix083 PMC5447851 28575360

[12] Antila M, He Q, de Jong C et al *Bordetella holmesii* DNA is not detected in nasopharyngeal swabs from Finnish and Dutch patients with suspected pertussis. J Med Microbiol 2006; 55:1043–51. 16849724 10.1099/jmm.0.46331-0

[13] Winter K, Zipprich J, Harriman K et al Risk factors associated with infant deaths from pertussis: a case-control study. Clin Infect Dis 2015; 61:1099–106. 26082502 10.1093/cid/civ472

[14] Tiwari TSP, Baughman AL, Clark TA First pertussis vaccine dose and prevention of infant mortality. Pediatrics 2015; 135:990–9. 25941302 10.1542/peds.2014-2291

[15] Paddock CD, Sanden GN, Cherry JD et al Pathology and pathogenesis of fatal *Bordetella pertussis* infection in infants. Clin Infect Dis 2008; 47:328–38. 18558873 10.1086/589753

[16] Berger JT, Carcillo JA, Shanley TP et al Critical pertussis illness in children: a multicenter prospective cohort study. Pediatr Crit Care Med 2013; 14:356–65. 23548960 10.1097/PCC.0b013e31828a70fe PMC3885763

[17] Sawal M, Cohen M, Irazuzta JE et al Fulminant pertussis: a multi-center study with new insights into the clinico-pathological mechanisms. Pediatr Pulmonol 2009; 44:970–80. 19725100 10.1002/ppul.21082

[18] Muloiwa R, Dube FS, Nicol MP, Zar HJ, Hussey GD Incidence and diagnosis of pertussis in South African children hospitalized with lower respiratory tract infection. Pediatr Infect Dis J 2016; 35:611–6. 26967813 10.1097/INF.0000000000001132

[19] Jones CE, Naidoo S, De Beer C, Esser M, Kampmann B, Hesseling AC Maternal HIV infection and antibody responses against vaccine-preventable diseases in uninfected infants. JAMA 2011; 305:576–84. 21304083 10.1001/jama.2011.100

[20] Haberling DL, Holman RC, Paddock CD, Murphy TV Infant and maternal risk factors for pertussis-related infant mortality in the United States, 1999 to 2004. Pediatr Infect Dis J 2009; 28:194–8. 19209089 10.1097/INF.0b013e31818c9032

[21] Sheridan SL, Ware RS, Grimwood K, Lambert SB Reduced risk of pertussis in whole-cell compared to acellular vaccine recipients is not confounded by age or receipt of booster-doses. Vaccine 2015; 33:5027–30. 26297874 10.1016/j.vaccine.2015.08.021

[22] Fulton TR, Phadke VK, Orenstein WA, Hinman AR, Johnson WD, Omer SB Protective effect of contemporary pertussis vaccines: a systematic review and meta-analysis. Clin Infect Dis 2016; 62:1100–10. 26908803 10.1093/cid/ciw051 PMC4826451

[23] Zar HJ, Barnett W, Stadler A, Gardner-Lubbe S, Myer L, Nicol MP Aetiology of childhood pneumonia in a well vaccinated South African birth cohort: a nested case-control study of the Drakenstein Child Health Study. Lancet Respir Med 2016; 4:463–72. 27117547 10.1016/S2213-2600(16)00096-5 PMC4989125

[24] Loeffelholz M Towards improved accuracy of *Bordetella pertussis* nucleic acid amplification tests. J Clin Microbiol 2012; 50:2186–90. 22442315 10.1128/JCM.00612-12 PMC3405598

[25] Pittet LF, Posfay-Barbe KM *Bordetella holmesii*: still emerging and elusive 20 years on. Microbiol Spectr 2016; 4. 10.1128/microbiolspec.EI10-0003-2015 27227292

[26] Cox HC, Jacob K, Whiley DM et al Further evidence that the IS481 target is suitable for real-time PCR detection of *Bordetella pertussis*. Pathology 2013; 45:202–3. 23277179 10.1097/PAT.0b013e32835cc2d7

[27] Cherry JD, Tan T, Wirsing von König C-H et al Clinical definitions of pertussis: summary of a Global Pertussis Initiative roundtable meeting, February 2011. Clin Infect Dis 2012; 54:1756–64. 22431797 10.1093/cid/cis302 PMC3357482

[28] van den Brink G, Wishaupt JO, Douma JC, Hartwig NG, Versteegh FGA *Bordetella pertussis*: an underreported pathogen in pediatric respiratory infections, a prospective cohort study. BMC Infect Dis 2014; 14:526. 25267437 10.1186/1471-2334-14-526 PMC4261543

[29] Kline JM, Lewis WD, Smith EA, Tracy LR, Moerschel SK Pertussis: a reemerging infection. Am Fam Physician 2013; 88:507–14. 24364571

[30] Wymann MN, Richard J-L, Vidondo B, Heininger U Prospective pertussis surveillance in Switzerland, 1991–2006. Vaccine 2011; 29:2058–65. 21251904 10.1016/j.vaccine.2011.01.017

[31] Van Rie A, Wendelboe AM, Englund JA Role of maternal pertussis antibodies in infants. Pediatr Infect Dis J 2005; 24:S62–5. 15876928 10.1097/01.inf.0000160915.93979.8f

[32] Casano P, Odena MP, Cambra FJ, Martín JM, Palomeque A *Bordetella pertussis* infection causing pulmonary hypertension. Arch Dis Child 2002; 86:453. 10.1136/adc.86.6.453-a PMC1762999 12023188

[33] Hoppe JE Neonatal pertussis. Pediatr Infect Dis J 2000; 19:244–7. 10749468 10.1097/00006454-200003000-00014

[34] Winter K, Harriman K, Zipprich J et al California pertussis epidemic, 2010. J Pediatr 2012; 161:1091–6. 22819634 10.1016/j.jpeds.2012.05.041

[35] Kohn JL, Fischer AE, Marks HH Case fatality in infants and children with pertussis, 1942–1946. Pediatrics 1950; 5:840–52. 15417285

[36] Nicoll A, Gardner A Whooping cough and unrecognised postperinatal mortality. Arch Dis Child 1988; 63:41–7. 3126714 10.1136/adc.63.1.41 PMC1779359

[37] Crowcroft NS, Booy R, Harrison T et al Severe and unrecognised: pertussis in UK infants. Arch Dis Child 2003; 88:802–6. 12937105 10.1136/adc.88.9.802 PMC1719623

[38] Eberhardt CS, Blanchard-Rohner G, Lemaître B et al Maternal immunization earlier in pregnancy maximizes antibody transfer and expected infant seropositivity against pertussis. Clin Infect Dis 2016; 62:829–36. 26797213 10.1093/cid/ciw027 PMC4787611

