# Supplementary material for: Pertussis-Associated Pneumonia in Infants and Children From Low- and Middle-Income Countries Participating in the PERCH Study
Source: Clin Infect Dis. 2016 Nov 2;63(Suppl 4):S187–96. doi: 10.1093/cid/ciw546 (PMC5106621; doi:10.1093/cid/ciw546)
Supplement: Supplementary Data [file supp_63_suppl-4_S187__index.html]

Supplementary Data 

# Pertussis-Associated Pneumonia in Infants and Children From Low- and Middle-Income Countries Participating in the PERCH Study

## Supplementary Data

Supplementary Data

- Supplementary Data - Docx file
