# Supplementary material for: Pertussis-Associated Pneumonia in Infants and Children From Low- and Middle-Income Countries Participating in the PERCH Study
Source: Clin Infect Dis. 2016 Nov 2;63(Suppl 4):S187–96. doi: 10.1093/cid/ciw546 (PMC5106621; doi:10.1093/cid/ciw546)
Supplement: Supplementary Data [file supp_ciw546_ciw546supp.docx]

**SUPPLEMENTARY MATERIALS**

**Supplementary Figure 1.** ***B. pertussis* nasopharyngeal (NP)/oropharyngeal (OP) swab PCR density, positive cases and controls 1-5 months of age**


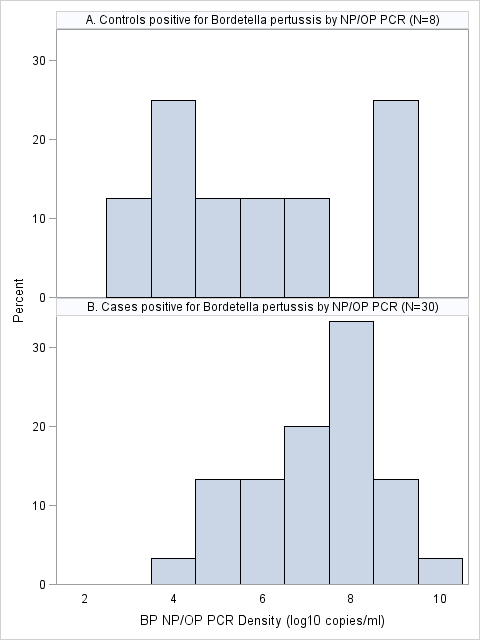


**Supplementary Table 1a. Characterization of sites based on pertussis vaccine formulation, schedule and availability of pertussis vaccine dosing data among cases aged 1-5 months**

| **Site** | **Pertussis Vaccine Formulation** | **Pertussis Vaccine Schedule** | **Age 1-5 months** | | | |
| --- | --- | --- | --- | --- | --- | --- |
|  |  |  | **Available Pertussis Vaccine Dosing Data^a^** | | **Available Pertussis Vaccine Immunization Records^b^** | |
|  |  |  | **Cases**  **N (%)** | **Controls**  **N (%)** | **Cases**  **N (%)** | **Controls**  **N (%)** |
| **Kenya** | DTP-Hib-HepB (Penta) | 6, 10, 14 weeks | 208 (99.5) | 234 (100.0) | 184 (88.5) | 186 (79.5) |
| **Gambia** | DTP-Hib-HepB (Penta) | 2, 3, 4 months | 249 (96.1) | 195 (98.0) | 245 (98.4) | 193 (99.0) |
| **Mali** | DTP-Hib-HepB (Penta) | 6, 10, 14 weeks | 303 (98.7) | 247 (100.0) | 125 (41.3) | 201 (81.4) |
| **Zambia** | DTP-Hib-HepB (Penta) | 6, 10, 14 weeks | 312 (95.4) | 308 (99.4) | 302 (96.8) | 305 (99.0) |
| **South Africa** | DTaP-Hib-IPV (Pentaxim)^c^ | 6, 10, 14 weeks | 444 (96.9) | 351 (96.2) | 442 (99.6) | 346 (99.1) |
| **Thailand** | DTP, DTP-HepB, DTP-Hib-HepB (Penta) | 2, 4, 6 months | 36 (94.7) | 90 (98.9) | 36 (100.0) | 89 (98.9) |
| **Bangladesh** | DTP, DTP-Hib-HepB (Penta) | 6, 10, 14 weeks | 135 (99.3) | 221 (100.0) | 119 (88.1) | 217 (98.2) |

^a^By maternal report or records; records defined as vaccination card or medical record.

^b^ Restricted to children with available pertussis vaccine dosing data.

^c^ DTP-Hib also available.

**Supplementary Table 1b. Coverage with three pertussis vaccines in the community, controls aged 9-18 months**

|  | **Age 9 – 18 months** | | | |
| --- | --- | --- | --- | --- |
|  | **Available Pertussis Vaccine Dosing Data** ^a^ | **Available Pertussis Vaccine Immunization Records^c^** | **Proportion Vaccinated^b^** | |
|  |  |  | **Among controls with available pertussis vaccine dosing data** | **Among controls with available pertussis vaccine immunization records** |
| **Site** | **Cases**  **N (%)** | **Controls**  **N (%)** | **Cases**  **N (%)** | **Controls**  **N (%)** |
| **Kenya** | 228 (98.3) | 145 (63.6) | 211 (92.5) | 133 (91.7) |
| **Gambia** | 154 (96.9) | 154 (100) | 136 (88.3) | 136 (88.3) |
| **Mali** | 165 (100) | 100 (60.6) | 148 (89.7) | 92 (92.0) |
| **Zambia** | 170 (98.3) | 168 (98.8) | 147 (86.5) | 145 (86.3) |
| **South Africa** | 237 (92.6) | 236 (99.6) | 172 (72.6) | 172 (72.9) |
| **Thailand** | 205 (99.5) | 202 (98.5) | 196 (95.6) | 193 (95.5) |
| **Bangladesh** | 207 (100) | 204 (98.6) | 200 (96.6) | 197 (96.6) |

^a^By maternal report or records; records defined as vaccination card or medical record.

^b^Proportion vaccinated defined as 3 or more doses of pertussis vaccine.

^c^Restricted to children with available pertussis vaccine dosing data.

**Supplementary Table 2. Characteristics of pertussis positive controls aged 1-5 months**

| **Site** | **Age (weeks)** | **Days of cough** | **Runny nose** | **Tachypnea** | **Vomiting** | **Fever** | **HIV status** | **No. pertussis vaccine doses^a^** |
| --- | --- | --- | --- | --- | --- | --- | --- | --- |
| **Mali** | 17 | 3 | Yes | Yes | Yes | No | Negative | 2 |
| **Mali** | 25 | 5 | Yes | No | No | Yes | Negative | 3 |
| **South Africa** | 4 | N/A | - | No | - | No | Negative | 0 |
| **South Africa** | 7 | N/A | No | No | No | No | Negative | 0 |
| **South Africa** | 9 | N/A | No | Yes | No | No | Negative | 1 |
| **South Africa** | 10 | N/A | No | Yes | No | No | Negative | 1 |
| **Thailand** | 9 | N/A | No | Yes | No | No | Negative | 0 |
| **Thailand** | 20 | N/A | No | No | No | Yes | Negative | 1 |

N/A, not applicable (i.e., no cough reported). -, missing data.

^a^ Number of pertussis vaccine doses received at 2 weeks prior to enrollment, or 2 weeks prior to start of cough.

**Supplementary Table 3**. **Characteristics of fatal *B. pertussis* positive cases aged 1-59 months**

| **Site** | **Age (weeks)** | **No. pertussis vaccine doses^a^** | **Severe/ very severe** | **HIV**  **(exposure/ infant status)** | **WBC 10^3^/µL** | **Lymph 10^3^/µL** | **Days of cough** | **CXR+^b^** | **Weight for age^c^** | **Days from admit to death** | **Pathogens detected** | | | |
| --- | --- | --- | --- | --- | --- | --- | --- | --- | --- | --- | --- | --- | --- | --- |
|  |  |  |  |  |  |  |  |  |  |  | **No.** | **IS PCR** | **NP/OP PCR** | **Blood culture** |
| **Ages 1-5 months** | | | | | | | | | | | | | | |
| **M** | 25 | 3 | S | Exposed/  Negative | 14.2 | 5.7 | 15 | Yes | Sev | 4 | 4 | N/A | Adeno, CMV, Para 3, *S.pneu* | None |
| **Z** | 13 | 0 | VS | Unexposed/ Negative | 66.4 | 40.3 | 30 | Yes | Sev | 18 | 4 | N/A | CMV, *H.inf*, *M.cat*, Rhino | None |
| **SA** | 13 | 1 | VS | Exposed/ Positive | 52.4 | 31.4 | 8 | Yes | Mod | 10 | 6 | CMV, *H.inf*, Para 1, PCP, Rhino, *S.aur* | CMV,  *H. inf,* Para 1, PCP, Rhino,  *S. aur* | None |
| **SA** | 7 | 0 | S | Unexposed/ Negative | 70 .0 | 30.8 | 3 | Yes | Norm | 1 | 6 | *M.cat*, Para 1, Rhino, *S.aur S.pneu,* | HBOV, *M.cat*, Para 1, Rhino, *S.pneu*, | None |
| **SA** | 7 | 0 | VS | Unexposed/ Negative | 47.9 | 27.8 | 4 | Yes | Norm | 6 | 6 | *H.inf, M.cat, S.pneu, S.aur* | CMV, PCP, *S.pneu, S.aur* | None |

| **Ages ≥6 months** | | | | | | | | | | | | | | |
| --- | --- | --- | --- | --- | --- | --- | --- | --- | --- | --- | --- | --- | --- | --- |
| **M** | 66 | 3 | VS | Unconfirmed | 30.6 | 15.6 | 7 | UNK | Norm | 1 | 6 | N/A | CMV, *H.inf*,  *M.cat*,  *S.pneu*, Rhino | *S. pneu* |
| **SA** | 44 | 0 | S | Unconfirmed exposure/ Negative | 24.7 | 12.1 | 2 | Yes | Norm | 4 | 2 | *S. pneu*, Rhino | *S. pneu*, Rhino | None |
| **Z** | 27 | 1 | S | Exposed/ Positive | 12.2 | 3.8 | 7 | UNK | Mod | 1 | 3 | N/A | CMV, *H.inf*, S.*pneu* | None |

Abbreviations: M=Mali, Z=Zambia, SA=South Africa; S: Severe, VS: Very Severe, Neg: Negative, Pos: Positive; UNK=unknown; N/A: not available.

Organisms: Adeno = adenovirus, CMV = cytomegalovirus, *H.inf* = *Hemophilus influenzae* (non-type b), HBOV = Human Bocavirus, *M.cat* = *Moraxella catarrhalis*, Para 1 = Parainfluenza type 1; Para 3 = Parainfluenza type 3, PCP = *Pneumocystis jirovecii,* Rhino = Rhinovirus, *S.pneu* = *Streptococcus pneumoniae*, *S.aur* = *Staphylococcus aureus*.

^a^ Number of DTP doses received ≥2 weeks before enrollment.

^b^ CXR+ defined as consolidation and/or other infiltrate. Two cases missing chest x-ray results.

^c^ Definitions for weight for age: Sev=Severe <-3 SDs; Mod=Moderate >=-3 SDs and <-2 SDs; Norm=Normal >=-2 SDs and <=+5 SDs.

Restricting to sites with fatal cases (Zambia, Mali and South Africa), chest x-ray positivity (p=0.023, Fisher’s) and elevated WBC (p=0.058, aOR, adjusted for site) were associated with mortality among pertussis-positive case

**Supplementary Table 4**: **Association of clinical, laboratory, and radiographic findings with pertussis status among cases and controls aged 6-59 months at sites with at least one positive case^a^**

|  | **Pertussis positive**  **(N=13)** | **Pertussis negative (N=2266)** | **Pertussis positive vs. negative** | |
| --- | --- | --- | --- | --- |
|  | **N (%)** | **N (%)** | **aOR^b^** | **p-value^b^** |
| **Runny nose, by report** | 4 (30.8) | 980 (43.3) | 0.65 | 0.51 |
| **Cough^c^** | 12 (92.3) | 2186 (96.5) | 0.41 | 0.42 |
| **Cough > 7 days** | 5 (45.5) | 310 (14.3) | 4.66 | 0.015 |
| **Cough > 14 days** | 0 (0.0) | 85 (3.9) | -- | -- |
| **Duration of cough, days, median (IQR) ^d^** | 3 (2-7) | 3 (2-4) | -- | 0.8859 |
| **Fever^c^** | 10 (76.9) | 1905 (84.1) | 0.75 | 0.67 |
| **Vomiting^c^** | 4 (30.8) | 495 (21.8) | 1.48 | 0.52 |
| **Unable to feed^c^** | 5 (38.5) | 245 (10.8) | 4.50 | 0.014 |
| **Tachypnea^e^** | 9 (69.2) | 1933 (85.5) | 0.40 | 0.14 |
| **Hypoxia^f^** | 7 (53.8) | 750 (33.2) | 1.60 | 0.47 |
| **Stridor** | 0 (0.0) | 58 (2.6) | -- | -- |
| **Grunting** | 4 (30.8) | 375 (16.7) | 3.12 | 0.13 |
| **Nasal flaring** | 9 (69.2) | 1257 (55.6) | 1.21 | 0.78 |
| **Deep breathing** | 5 (38.5) | 552 (24.4) | 2.8 | 0.084 |
| **Audible wheeze** | 2 (15.4) | 401 (17.7) | 1.4 | 0.73 |
| **CXR positive** | 3 (30.0) | 1075 (50.4) | 0.35 | 0.13 |
| **WBC (1000 cells/µL), median (IQR)** | 15.5 (12.2-25.7) | 13.6 (9.8-17.9) | -- | 0.048 |
| **WBC > 20 (1000 cells/µL)** | 5 (41.7) | 374 (17.5) | 3.3 | 0.049 |
| **Lymphocyte count (1000 cells/µL), median (IQR)** | 6.0 (4.2-9.1) | 4.4 (2.9-6.7) | -- | 0.047 |
| **Lymphocyte count > 10 (1000 cells/µL)** | 2 (16.7) | 174 (8.2) | 2.2 | 0.31 |

Abbreviations: aOR, adjusted odds ratio; WBC, White blood cell; IQR, interquartile range; NP/OP, nasopharyngeal/oropharyngeal.

^a^ Analysis restricted to sites with at least 1 pertussis-positive case (Kenya, The Gambia, Zambia, Mali, South Africa and Bangladesh).

^b^ Odds ratios and p-values from logistic regression models adjusted for site. After adjusting the WBC and lymphocyte analyses for age, there was a minor change in the ORs and p-values (WBC OR 3.3, P=0.043; Lymphocyte 2.3, P=0.307).

^c^ By history and/or physical exam; 5 of the 10 fevers (50%) were medically documented.

^d^ Restricted to children with cough by history and/or physical examination. Duration in days.

^e^ Respiratory rate >60 breaths/minute if aged <2 months, respiratory rate >50 breaths/minute if aged 2-5 months.

^f^ A child was considered to be hypoxic if 1) a room air pulse-oximetry reading indicated oxygen saturation <90% at the two sites at elevation (Zambia and South Africa) or <92% at all other sites, or 2) a room air oxygen saturation reading was not available and the child was on oxygen.

**APPENDIX: Sensitivity analysis estimating pertussis case fatality accounting for cases without PCR test and those for whom 30-day vital status was not ascertained**

At the African sites, there were 13 cases among children 1-5 months of age who were not tested for pertussis; of these 5 died (38.5%). Of the 35 pertussis-positive cases discharged alive, 21 (60.0%) had 30-day vital status recorded with no additional deaths documented; vital status ascertainment was lower than for pertussis-negative cases (84.6% of 1375 discharged alive had 30-day vital status). Under assumptions about the pertussis status of cases and deaths for those cases missing data, the CFR could be as low as 10.4% and as high as 53.0%. The former occurs when we assume no deaths among the 14 pertussis-positive cases discharged alive who were missing 30-day vital status data, and assume the cases not tested for pertussis who survived (n=8) were pertussis positive while those who died (n=5) were negative. The latter occurs when we assume that all 14 pertussis-positive cases discharged alive, but without documented vital status at 30-day follow-up had in fact died by that point, and that among cases not tested for pertussis (n=13), the cases who died (n=5) were pertussis positive while all remaining cases (n=8) were negative. With no pertussis-positive cases among children <6 months of age in the Thailand and Bangladesh sites, no pertussis mortality analyses were possible.
